# Lactate Dehydrogenase (LDH) Response to First-Line Treatment Predicts Survival in Metastatic Breast Cancer: First Clues for a Cost-Effective and Dynamic Biomarker

**DOI:** 10.3390/cancers11091243

**Published:** 2019-08-24

**Authors:** Giacomo Pelizzari, Debora Basile, Silvia Zago, Camilla Lisanti, Michele Bartoletti, Lucia Bortot, Maria Grazia Vitale, Valentina Fanotto, Serena Barban, Marika Cinausero, Marta Bonotto, Lorenzo Gerratana, Mauro Mansutti, Francesco Curcio, Gianpiero Fasola, Alessandro Marco Minisini, Fabio Puglisi

**Affiliations:** 1 Department of Medical Oncology, Centro di Riferimento Oncologico di Aviano (CRO), IRCCS, 33081 Aviano (PN), Italy; 2 Department of Medicine (DAME), University of Udine, 33100 Udine, Italy; 3 Clinical Pathology Institute, ASUIUD University Hospital of Udine, 33100 Udine, Italy; 4 Clinical Pathology, Hospital “Santa Maria degli Angeli”, 33170 Pordenone, Italy; 5 Department of Oncology, ASUIUD University Hospital of Udine, 33100 Udine, Italy

**Keywords:** metastatic breast cancer, lactate dehydrogenase, serum biomarker, LDH, monitoring metastatic breast cancer

## Abstract

Background: Elevated plasmatic lactate dehydrogenase (LDH) levels are associated with worse prognosis in various malignancies, including metastatic breast cancer (MBC). Nevertheless, no data are available on the prognostic role of LDH as a dynamic biomarker during first-line treatment in unselected MBC. Methods: We reviewed data of 392 women with MBC to evaluate the association between LDH variation after 12 weeks of first-line treatment and survival. The prognostic impact was tested by multivariate Cox regression analysis. Results: Plasmatic LDH was confirmed as an independent prognostic factor in MBC. Patients who maintained elevated LDH levels after 12 weeks of first-line treatment experienced worse progression-free survival (PFS, HR 2.88, 95% CI: 1.40–5.89, *p* = 0.0038) and overall survival (OS, HR 2.61, 95% CI 1.16–5.86, *p* = 0.02) compared to patients with stable normal LDH levels, even after adjustment for other prognostic factors. Notably, LDH low-to-high variation emerged as an unfavorable prognostic factor for PFS (HR 3.96, 95% CI 2.00–7.82, *p* = 0.0001). Conclusions: Plasmatic LDH and its variation during first-line treatment predict PFS and OS in MBC, providing independent prognostic information. It would be worthwhile to prospectively evaluate the association between LDH variation and therapeutic benefit in MBC, and explore how it may affect treatment strategies.

## 1. Introduction

Breast cancer (BC) is the most common cancer among women and the second leading cause of cancer-related death [1]. About 6% of all breast tumors present with distant metastases at diagnosis, and 30% of patients with early BC will experience local or distant recurrence [2]. BC is a heterogeneous disease, including distinct subgroups with different prognosis based on histological and molecular features [3]. In clinical practice, the expression of the estrogen receptor (ER), progesterone receptor (PR), and the human epidermal growth factor receptor 2 (HER2) identifies three main subgroups: Luminal or hormone receptor positive (HR-positive) BC, HER2-positive BC, and triple negative breast cancer (TNBC) [4]. Despite new treatments and improved standard of care, metastatic breast cancer (MBC) remains an incurable disease with a median survival of about 34 months, even if it varies significantly among and within the subgroups [5]. Therefore, it is essential to identify tumor- and patient-related factors able to predict aggressive biological behavior and treatment resistance. Recently, several studies evaluated novel circulating biomarkers in BC, including inflammatory factors [6], exosomes [7], circulating tumor DNA (ctDNA) [8], and circulating tumor cells (CTC) [9]. However, even routinely used biomarkers (e.g., the neutrophil-to-lymphocyte ratio [10], lactate dehydrogenase (LDH) [11], alkaline phosphatase (ALP) [12]) provide additional information on tumor biology and should be further evaluated for their prognostic relevance.

LDH is a ubiquitous enzyme that plays a central role in anaerobic glycolysis, as it catalyzes the reversible conversion of pyruvate into lactate [13]. LDH comprises a family of six tetrameric isoenzymes [14,15] with a tissue-specific expression regulated by both physiological and pathological conditions. The *LDHA* gene expression is upregulated in several types of cancers, especially in rapidly growing tumors, to maintain glycolysis as an alternative source of energy during hypoxic stress and subsequent high LDH level in cytoplasmic compartment. Notably, different extracellular factors, such as hormones, growth factors, and cytokines can regulate LDH expression by receptor-dependent and -independent intracellular signaling pathways (e.g., cAMP Response Element-Binding protein (CREB), Hypoxia-Inducible Factor-1 (HIF-1), and c-Myc) [15]. Beyond its role in regulating cellular metabolism, LDH is a well-known marker of tissue damage. Many pathological conditions, including cancer, present with LDH elevation due to acute cell death or necrosis. Moreover, high plasmatic LDH levels influence tumor progression and metastatic spread with a negative impact on outcome in various cancer types [16,17,18,19,20,21,22,23,24,25].

The prognostic role of plasmatic LDH levels has been investigated in BC as well. The first piece of evidence dates back to the late 1990s and early 2000s when three extensive studies found that elevated plasmatic LDH levels were associated with poor outcome in MBC patients [26,27,28]. High plasmatic LDH levels were also proven to be significantly associated with increased risk of disease recurrence and death [12,29]. Notably, a recent meta-analysis confirmed these findings in both MBC and early BC [11]. 

Nevertheless, no data are available on the prognostic role of LDH dynamic response to first-line treatment in unselected MBC patients. Thus, we conducted an exploratory study to identify the prognostic impact of plasmatic LDH variation after 12 weeks of first-line treatment on both progression-free survival (PFS) and overall survival (OS) in MBC.

## 2. Results

### 2.1. Patient’s Characteristics

A consecutive series of 392 women with MBC were included in the analysis, 219 with a plasmatic LDH evaluation at baseline. The median age was 62 years (range 29–88), with 42.9% of patients older than 65 years and 10.7% younger than 45 years. Invasive ductal carcinoma was the most common histology (80.4% of cases), and post-menopausal women accounted for 59.4% of patients. Approximately 60.5% of patients had HR-positive tumors (11.2% were luminal A, 38.3% luminal B, and 11.0% luminal HER2-positive; see Section 4.2. for classification details), 8.7% had HR-negative/HER2-positive disease, and 9.4% TNBC. At MBC diagnosis, nearly half of the patients presented with a single metastatic site, and about 20% had three or more localizations. Bone metastases were detected in half of the cases (20% of patients had a bone-only disease), while patients with liver, lung, or central nervous system localizations (CNS) were about 25%, 28%, and 6.4%, respectively. Overall, nearly 60% of patients received chemotherapy as first-line treatment, and the remaining 40% received hormonal therapy. Additional baseline clinical and pathologic characteristics of patients are listed in Table 1.

### 2.2. Prognostic Role of Pre-Treatment Plasmatic LDH

**Figure 1:**
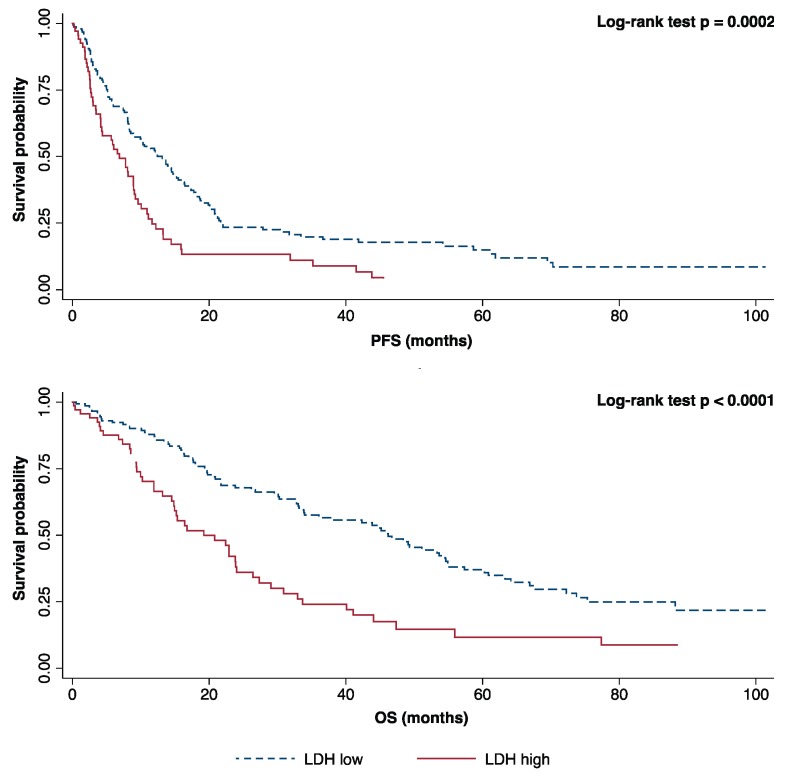
Kaplan–Meier curves for progression-free survival (PFS) and overall survival (OS) according to baseline lactate dehydrogenase (LDH).

After a median follow-up of 52.77 months, median OS was 30.87 months (25–75th percentile: 13.50–62.80), and median PFS was 9.21 months (25–75th percentile: 3.95–20.70). At baseline, 31.5% of evaluable patients (69/219) had elevated pre-treatment LDH levels according to the centralized laboratory cut-off (>480 UI/L). Through univariate analyses, baseline elevated plasmatic LDH emerged as an unfavorable prognostic factor in terms of PFS and OS. More specifically, patients with baseline elevated LDH experienced shorter median PFS (6.87 vs. 13.12 months, HR 1.81, 95% CI: 1.31–2.51, *p* = 0.0003) and OS (19.23 vs. 46.19 months, HR 2.23, 95% CI: 1.55–3.19, *p* < 0.0001) compared to patients with normal LDH (Figure 1). The prognostic role of LDH plasma levels was also confirmed when evaluated as a continuous variable for both PFS (*p* = 0.0002) and OS (*p* < 0.0001).

These findings were confirmed for both PFS (HR 1.51, 95% CI: 1.02–2.26, *p* = 0.039) and OS (HR 1.64, 95% CI: 1.05–2.55, *p* = 0.027) after multivariate adjustment for molecular profiles, Eastern Cooperative Oncology Group Performance Status (ECOG PS), baseline ALP level, number of metastatic sites, central nervous system (CNS), and liver and bone localizations (Table 2 and Table 3).

**Figure 2:**
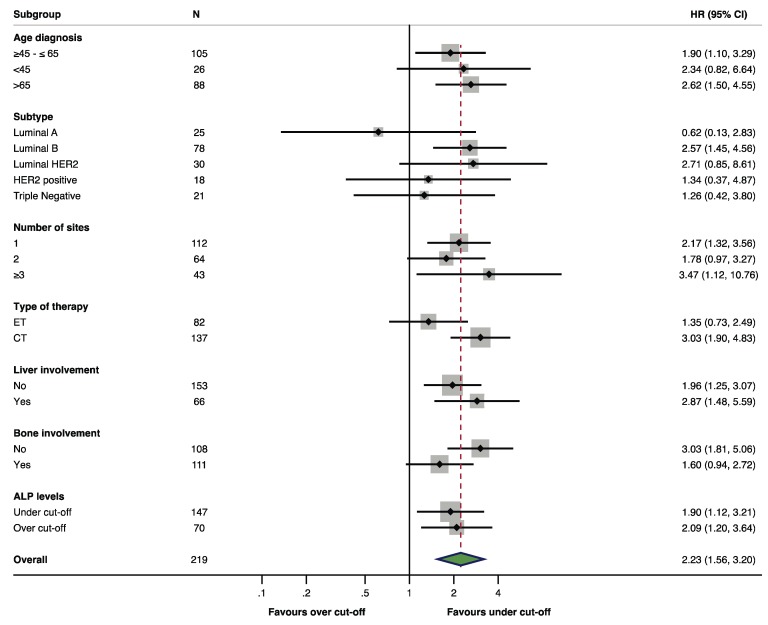
Subgroup analysis of OS in patients with baseline elevated LDH vs. normal LDH level.

The role of LDH as an adverse prognostic factor was consistent in all examined subgroups: Age, profile, number of metastatic sites, type of first-line treatment (hormonal therapy or chemotherapy), baseline ALP level, and liver and bone involvement (Figure 2). Aside from baseline LDH level, other independent prognostic factors for PFS were triple negative profile (HR 2.81, 95% CI: 1.44–5.48, *p* = 0.002) and ECOG PS (2 vs. 0, HR 2.45, 95% CI: 1.18–5.07, *p* = 0.015), while for OS, they were luminal B profile (HR 2.26, 95% CI: 1.16–4.38, *p* = 0.015), triple negative profile (HR 7.19, 95% CI: 3.11–16.58, *p* < 0.0001), ECOG PS (1 vs. 0, HR 1.88, 95% CI: 1.18–2.99, *p* = 0.007), tumor burden (2 vs. 1 localizations, HR 2.04, 95% CI: 1.20–3.46, *p* = 0.008), and CNS localizations (HR 22.05, 95% CI: 4.38–110.94, *p* = 0.002). The complete Cox regression model is reported in Table 2 and Table 3.

### 2.3. Prognostic Role of Plasmatic LDH Response during First-Line Treatment.

LDH value after 12 weeks of first-line treatment was available in 126 patients (32%). Among them, 54.7% had stable low LDH levels, 15.0% had stable high levels, and in approximately 30% of cases, LDH levels changed over time across the upper normal limit (12% had a drop under the upper normal limit, while 18.2% had a rise over the upper normal limit). 

**Figure 3:**
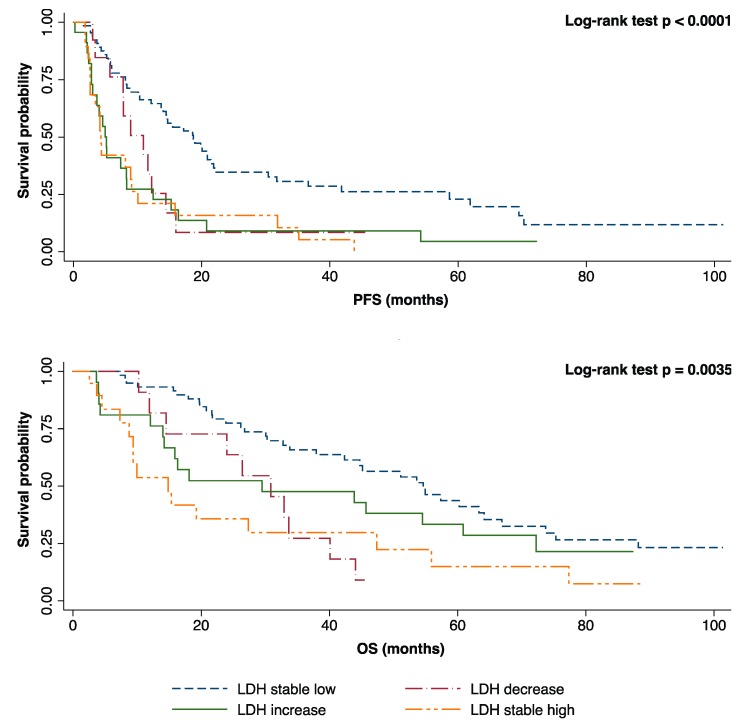
Kaplan–Meier curves for PFS and OS according to plasmatic LDH variation after 12 weeks of first-line treatment.

According to plasmatic LDH variation, we were able to detect significant differences of both median PFS (stable low levels: 18.71 months, high-to-low levels: 10.92 months, low-to-high levels: 5.13 months, stable high levels: 4.27 months, *p* < 0.0001) and median OS (stable low levels: 54.64 months, high-to-low levels: 30.87 months, low-to-high levels: 29.49 months, stable high levels: 14.83 months, *p* < 0.0001) (Figure 3 and Table 4).

The prognostic relevance of LDH response to first-line treatment was then assessed using a Cox regression multivariate model. Stable elevated LDH levels after 12 weeks of first-line treatment was confirmed as an independent negative prognostic factor for both PFS (HR 2.88, 95% CI: 1.40–5.89, *p* = 0.0038) and OS (HR 2.61, 95% CI 1.16–5.86, *p* = 0.02) after multivariate adjustment for molecular profile, ECOG PS, number of metastatic sites, CNS, liver, bone localizations, and plasmatic ALP variation at 12 weeks. Moreover, a rise in plasmatic LDH levels after 12 weeks of first-line treatment (low-to-high variation) also emerged by multivariate analysis as an independent negative prognostic factor for PFS (HR 3.96, 95% CI 2.00–7.82, *p* = 0.0001) with a trend for worse OS (HR 2.02, 95% 0.89–4.56, *p* = 0.08). The complete Cox regression model is reported in Table 5.

## 3. Discussion

Many studies reported elevated plasmatic LDH levels to be associated with poor outcomes in various tumors [30]. A recent meta-analysis, including 76 studies conducted in patients with several cancer types, confirmed that high LDH plasmatic levels were associated with shorter PFS and OS [31]. Although the prognostic role of LDH in cancer is well-established, the underlying biological mechanisms are still unclear, and some possible explanations have been hypothesized. Firstly, high LDH plasmatic concentrations sustain anaerobic metabolism during tumor growth and metastatic spread, supporting the energetic requirements in hypoxic conditions [32]. Secondly, LDH exerts an inflammatory action on tumor microenvironment, activating interleukin (IL)-23 and IL-17 and modulating the activity of arginase I. It inhibits CD8+ T lymphocytes and natural killer (NK) activation, allowing cancer cells to evade immune response [33]. Moreover, high LDH levels promote tumor angiogenesis, cell migration, and metastatization by inhibiting the degradation of HIF-1 alpha and increasing the production of vascular endothelial growth factor (VEGF) [34]. Thirdly, preliminary evidence suggests that increased *LDHA* expression and lactate overproduction might also play a role in drug resistance [35]. 

The present study investigated the prognostic impact of plasmatic LDH levels on survival outcomes in MBC patients at first-line treatment. 

Approximately 31% of evaluated patients had high baseline LDH levels and about 32% had an LDH variation during first-line treatment. In particular, 15% of patients had a stable high LDH and 18% had a low-to-high variation. 

The results confirmed that elevated baseline LDH levels were independently associated with shorter PFS (6.87 vs. 13.12 months, adjusted HR 1.51, 95% CI: 1.02–2.26, *p* = 0.039) and OS (19.23 vs. 46.19 months, adjusted HR 1.64, 95% CI: 1.05–2.55, *p* = 0.027). These data were also confirmed when LDH plasma levels were evaluated as a continuous variable (PFS, OS), so our results were not dependent on the pre-specified cut-off for normal LDH plasmatic concentrations. To the best of our knowledge, this is the first study to demonstrate that LDH changes during first-line treatment significantly impact both PFS and OS in unselected MBC patients. Specifically, patients with elevated baseline plasmatic LDH who maintained high LDH levels after 12 weeks of first-line treatment experienced worse PFS and OS compared to patients with stable normal LDH levels, even after adjustment for other prognostic factors (HR 2.88, 95% CI: 1.40–5.89, *p* = 0.0038 and HR 2.61, 95% CI 1.16–5.86, *p* = 0.02 for OS and PFS, respectively). Interestingly, since elevated plasmatic LDH levels may also reflect the presence of high tumor burden, bone localizations, liver metastases, and ALP levels variations, it is noteworthy that their prognostic value was maintained after including these covariates in the multivariate Cox regression model.

Additionally, plasmatic LDH elevation during first-line treatment emerged as an independent prognostic factor for PFS (HR 3.96, 95% CI 2.00–7.82, *p* = 0.0001) with a trend for OS (HR 2.02, 95% 0.89–4.56, *p* = 0.08). In accordance with our findings, a recent study conducted in TNBC patients confirmed that LDH changes after two cycles of first-line chemotherapy correlate with objective response rate and PFS [36].

Therefore, LDH can predict survival in patients with MBC and provides independent and dynamic prognostic information during first-line treatment. Given our results, patients with stable high LDH levels or with LDH elevation during first-line therapy may be monitored more frequently for disease progression, as they might experience shorter PFS. Conversely, patients with stable normal LDH levels will experience prolonged PFS and OS. Nevertheless, since these findings are not prospectively validated, LDH variation must not be considered an indirect proof of tumor progression or response, even if it offers additional prognostic information. 

In our study, LDH-A tissue expression was not tested. However, its relationship with plasmatic LDH may be useful to define whether LDH plasmatic elevation is primarily tumor-related or not, exploring the biological significance and the prognostic value of their concordance or discordance. According to previous studies, elevated tissue LDH-A expression is associated with elevated Ki-67, high proliferation rates, and CNS metastases in TNBC [37].

The main strength of our study is the identification of a dynamic, easy-to-use, inexpensive, and reproducible prognostic biomarker in patients with unselected MBC. However, this is a retrospective and single-center study. Thus, prospective and external validation is mandatory. Moreover, the LDH cut-off value for normality implemented in this study may differ in other centers; consequently, its reproducibility has to be confirmed. Lastly, we did not consider the potential interaction of several other non-neoplastic diseases (e.g., heart failure, anemia, hypothyroidism, autoimmune, and lung disorders), which might influence plasmatic LDH levels.

On the basis of these observations, it would be of great value to prospectively evaluate the potential correlation between LDH variation and response to treatment in MBC, and explore the prognostic role of this long-standing biomarker in the modern era of immunotherapy and targeted therapy.

## 4. Materials and Methods 

### 4.1. Study Design

This observational, retrospective, no-profit, monocentric cohort study examined data of 392 consecutive MBC patients treated between 2007 and 2017 at the Department of Oncology of the University Hospital of Udine (Italy). The study was conducted under the Declaration of Helsinki, and the Regional Ethics Committee approved the protocol (N° Protocol 14571 ratified in May 2018). Informed consent was obtained for the use of clinical data, rendered anonymous, for purposes of clinical research, epidemiology, training, and study of diseases. 

### 4.2. Data Source

Clinicopathological information and blood sample data were collected from electronic health records. We defined MBC subgroups as follows: Luminal A (ER or PR positive, HER2-negative, Ki-67 ≤ 14%), luminal B (ER or PR positive, HER2-negative, Ki-67 > 14%), luminal HER2 (HER2-positive and ER or PR positive), HER2-positive (ER and PR negative, HER2-positive), and triple negative (ER and PR negative, HER2-negative) [4]. 

### 4.3. Blood Sample Analysis

Serum LDH and ALP data were retrospectively evaluated. Blood samples data were eligible for review if performed within one month before first-line treatment administration (baseline pre-treatment sample) and after 12 weeks ± 1 week after first treatment dose (post-treatment sample). The quantitative determination of LDH and ALP was performed using the Roche Cobas 8000 c702 system (Roche Diagnostics, Indianapolis, IN, USA). The LDH and ALP cut-off value for normality was the normal upper limit (NUL) defined by the analytical system used (480 IU/L and 104 IU/L, respectively).

### 4.4. Statistical Analysis

The study was designed in order to explore the prognostic role of LDH response after 12 weeks of first-line treatment in unselected MBC, with a hierarchical design: The independent prognostic impact of plasmatic LDH was first evaluated at baseline and then for its variation at 12 weeks, using a multivariate Cox regression model for both PFS and OS with 95% confidence interval (95% CI). A two-sided *p* < 0.05 was considered statistically significant. The multivariate model included the following covariates: The molecular profile, ECOG PS, number of metastatic sites, CNS, liver and bone localizations, and plasmatic ALP levels (at baseline and its variation at 12 weeks). Baseline clinicopathological characteristics were summarized through descriptive analysis. OS was defined as the time elapsed between the start of first-line treatment and death or last follow-up. PFS was defined as the interval between the start of first-line treatment and disease progression or death for any cause. Differences in survival were tested by a log-rank test and represented by Kaplan–Meier survival curves. Statistical analysis was performed with STATA (StataCorp, www.stata.com (2015) Stata Statistical Software: Release 14.2. College Station, TX: StataCorp LP).

## 5. Conclusions

LDH is a routinely used biomarker with a well-established prognostic role in several solid tumors and hematological malignancies. Our study confirmed that LDH is an independent prognostic factor also in MBC and explored its value as a dynamic biomarker. To the best of our knowledge, this is the first study to demonstrate that LDH response to first-line treatment significantly impacts both PFS and OS in unselected MBC patients. If validated in prospective studies, LDH could represent a cost-effective biomarker to stratify patient’s prognosis, monitor treatment efficacy, and to implement treatment strategies in MBC. 

## Figures and Tables

**Table 1:** Baseline patients’ clinical and pathologic characteristics.

Characteristics		Number of Patients (Total = 392)	%
Age	<45 years	42	10.71
	45–65 years	182	46.43
	>65 years	168	42.86
Menopausal state	Pre-menopausal	114	29.08
	Post-menopausal	233	59.44
	Unknown	45	11.48
Histotype	Ductal	315	80.36
	Lobular	59	15.05
	Other	12	3.06
	Unknown	6	1.53
Profile	Luminal A	44	11.22
	Luminal B	150	38.27
	Luminal HER2	43	10.97
	HER2-positive	34	8.67
	Triple negative	37	9.44
	Unknown	84	21.43
ECOG PS	0	201	51.28
	1	150	38.26
	≥2	34	8.67
	Unknown	7	1.79
Number of metastatic sites	1	212	54.08
	2	104	26.53
	≥3	76	19.39
Site of metastases *	Bone	199	50.77
	Bone only	79	20.15
	Liver	99	25.26
	CNS	25	6.38
	Lung	110	28.06
	Lymph nodes	133	33.93
Firs-line treatment	Chemotherapy	231	58.93
	Hormonal therapy	161	41.07
Baseline LDH level	High ^1^	69	17.60
	Normal	150	38.27
	Unknown	173	44.13
Baseline ALP level	High ^2^	124	31.63
	Normal	245	62.50
	Unknown	23	5.87

Legend: CNS, Central Nervous System; LDH, Lactate dehydrogenase; ALP, alkaline phosphatase; ECOG PS, Eastern Cooperative Oncology Group Performance Status. ^1^ LDH > 480 IU/L; ^2^ ALP > 104 IU/L; * Patients may present more than one metastatic site.

**Table 2:** Baseline prognostic factors for PFS according to univariate and multivariate Cox model.

Covariates		Number of Patients	Univariate Analysis(HR, 95% CI)	*p*	Multivariate Analysis(HR, 95% CI)	*p*
Age	<45 years	42	0.80 (0.54–1.17)	0.25		
	45–65 years	182	Ref.	-		
	>65 years	168	0.95 (0.75–1.20)	0.68		
Profile	Luminal A	44	Ref.	-	Ref.	-
	Luminal B	150	1.27 (0.87–1.87)	0.20	1.10 (0.66–1.84)	0.68
	Luminal HER2	43	0.73 (0.44–1.19)	0.21	0.58 (0.31–1.10)	0.09
	HER2-positive	34	1.17 (0.71–1.92)	0.52	0.92 (0.44–1.92)	0.84
	Triple negative	37	3.19 (1.96–5.17)	<0.0001	2.81 (1.44–5.48)	0.002
ECOG PS	0	201	Ref.	-	Ref.	-
	1	150	1.25 (0.98–1.58)	0.06	1.35 (0.90–2.02)	0.13
	≥2	34	1.70 (1.12–2.59)	0.01	2.45 (1.18–5.07)	0.01
Number of metastatic sites	1	212	Ref.	-	Ref.	-
	2	104	1.35 (1.04–1.75)	0.02	1.51 (0.97–2.35)	0.06
	≥3	76	0.97 (0.71–1.34)	0.89	0.61 (0.35–1.04)	0.07
Site of metastases *	Bone	199	0.93 (0.74–1.16)	0.54	1.07 (0.71–1.61)	0.73
	Liver	99	1.14 (0.88–1.47)	0.29	0.92 (0.58–1.47)	0.75
	CNS	25	1.38 (0.86–2.20)	0.17		
	Lung	110	0.90 (0.70–1.16)	0.44		
Baseline LDH level	High ^1^	69	1.81 (1.31–2.51)	0.0003	1.51 (1.02–2.26)	0.039
	Normal	150	Ref.	-	Ref.	-
Baseline ALP level	High ^2^	124	1.45 (1.14–1.85)	0.002	1.11 (0.73–1.66)	0.61
	Normal	245	Ref.	-	Ref.	-

Legend: CNS, Central Nervous System; LDH, Lactate dehydrogenase; ALP, alkaline phosphatase; ECOG PS, Eastern Cooperative Oncology Group Performance Status; Ref., Reference. ^1^ LDH cut-off: 480 IU/L; ^2^ ALP cut-off: 104 IU/L; * Patients may present more than one metastatic site.

**Table 3:** Baseline prognostic factors for OS according to univariate and multivariate Cox model.

Covariates		Number of Patients	Univariate Analysis(HR, 95% CI)	*p*	Multivariate Analysis(HR, 95% CI)	*p*
Age	<45 years	42	0.82 (0.52–1.29)	0.41		
	45–65 years	182	Ref.	-		
	>65 years	168	1.16 (0.89–1.53)	0.25		
Profile	Luminal A	44	Ref.	-	Ref.	-
	Luminal B	150	1.63 (1.01–2.62)	0.04	2.26 (1.16–4.38)	0.01
	Luminal HER2	43	1.14 (0.63–2.03)	0.65	1.73 (0.81–3.70)	0.15
	HER2-positive	34	1.61 (0.88–2.96)	0.12	1.24 (0.49–3.15)	0.64
	Triple negative	37	4.31 (2.45–7.59)	<0.0001	7.19 (3.11–16.5)	<0.0001
ECOG PS	0	201	Ref.	-	Ref.	-
	1	150	1.92 (1.45–2.55)	<0.0001	1.88 (1.18–2.99)	0.007
	≥2	34	2.61 (1.72–3.97)	<0.0001	1.76 (0.84–3.70)	0.13
Number of metastatic sites	1	212	Ref.	-	Ref.	-
	2	104	1.40 (1.03–1.89)	0.02	2.04 (1.20–3.46)	0.008
	≥3	76	1.36 (0.95–1.94)	0.08	0.70 (0.35–1.41)	0.32
Site of metastases *	Bone	199	0.95 (0.73–1.23)	0.74	1.19 (0.73–1.96)	0.47
	Liver	99	1.33 (1.00–1.78)	0.046	0.58 (0.68–1.93)	0.58
	CNS	25	2.72 (1.68–4.42)	<0.0001	22.05 (4.38–110.94)	0.0002
	Lung	110	1.10 (0.82–1.47)	0.51		
Baseline LDH level	High ^1^	69	2.22 (1.55–3.19)	<0.0001	1.64 (1.05–2.54)	0.027
	Normal	150	Ref.	-	Ref.	-
Baseline ALP level	High ^2^	124	1.84 (1.40–2.41)	<0.0001	1.48 (0.94–2.31)	0.08
	Normal	245	Ref.	-	Ref.	-

Legend: CNS, Central Nervous System; LDH, Lactate dehydrogenase; ALP, alkaline phosphatase; ECOG PS, Eastern Cooperative Oncology Group Performance Status; Ref., Reference. ^1^ LDH cut-off: 480 IU/L; ^2^ ALP cut-off: 104 IU/L; * Patients may present more than one metastatic site.

**Table 4:** OS and PFS according to plasmatic LDH variation after 12 weeks of first-line treatment.

LDH Variation ^1^	Number of Patients(Total = 126)	%	Median PFS(25–75th Percentile)	Median OS(25–75th Percentile)
Stable low	69	54.76	18.71 (8.09–58.65)	54.64 (26.76–88.18)
High-to-low	15	11.91	10.92 (7.76–14.43)	30.87 (14.53–40.08)
Low-to-high	23	18.25	5.13 (2.79–12.43)	29.49 (14.01–72.26)
Stable high	19	15.08	4.27 (2.60–10.06)	14.83 (8.78–47.38)

Legend: LDH, Lactate dehydrogenase. ^1^ LDH cut-off: 480 IU/L.

**Table 5:** LDH variation after 12 weeks of first-line treatment: Prognostic impact on PFS and OS according to multivariate Cox model.

Covariates		Number of Patients	Multivariate Analysis (HR, 95% CI) PFS	*p*	Multivariate Analysis (HR, 95% CI) OS	*p*
Profile	Luminal A	44	Ref.	-	Ref.	-
	Luminal B	150	0.88 (0.43–1.80)	0.73	1.39 (0.57–3.36)	0.46
	Luminal HER2	43	0.37 (0.17–0.81)	0.01	0.75 (0.30–1.86)	0.54
	HER2-positive	34	1.12 (0.41–3.03)	0.12	0.66 (0.18–2.40)	0.53
	Triple negative	37	2.90 (1.16–7.22)	0.02	7.81 (2.66–22.9)	0.0002
ECOG PS	0	201	Ref.	-	Ref.	-
	1	150	1.68 (0.90–3.14)	0.10	1.78 (0.84–3.79)	0.13
	≥2	34	4.19 (1.48–11.85)	0.006	2.29 (0.81–6.47)	0.11
Number of metastatic sites	1	212	Ref.	-	Ref.	-
	2	104	1.86 (0.92–3.73)	0.08	1.75 (0.79–3.88)	0.16
	≥3	76	0.71 (0.32–1.56)	0.40	0.67 (0.25–1.79)	0.43
Site of metastases *	Bone	199	1.46 (0.76–2.81)	0.25	2.35 (1.05–5.23)	0.036
	Liver	99	0.88 (0.44–1.76)	0.72	1.53 (0.71–3.31)	0.26
	CNS	25			1223.5 (42.5–35225.6)	<0.0001
ALP variation at 12 weeks ^1^	Stable low	20262	Ref.	-	Ref.	-
	High-to-low	44	0.82 (0.35–1.87)	0.63	0.62 (0.25–1.52)	0.30
	Low-to-high	13	0.88 (0.22–3.42)	0.86	2.39 (0.57–10.0)	0.23
	Stable high	62	0.98 (0.45–2.12)	0.96	1.24 (0.53–2.88)	0.60
LDH variation at 12 weeks ^2^	Stable low	69	Ref.	-	Ref.	-
	High-to-low	15	1.27 (0.50–3.23)	0.60	2.35 (0.82–6.77)	0.11
	Low-to-high	23	3.96 (2.00–7.82)	0.0001	2.02 (0.89–4.56)	0.08
	Stable high	19	2.88 (1.40–5.89)	0.003	2.61 (1.16–5.86)	0.02

Legend: CNS, Central Nervous System; LDH, Lactate dehydrogenase; ALP, alkaline phosphatase; Ref., Reference; ECOG PS, Eastern Cooperative Oncology Group Performance Status. ^1^ ALP cut-off: 104 IU/L; ^2^ LDH cut-off: 480 IU/L; * Patients may present more than one metastatic site.
